# Increased fatty acyl saturation of phosphatidylinositol phosphates in prostate cancer progression

**DOI:** 10.1038/s41598-019-49744-3

**Published:** 2019-09-13

**Authors:** Atsushi Koizumi, Shintaro Narita, Hiroki Nakanishi, Masaki Ishikawa, Satoshi Eguchi, Hirotaka Kimura, Shunsuke Takasuga, Mingguo Huang, Takamitsu Inoue, Junko Sasaki, Toshiaki Yoshioka, Tomonori Habuchi, Takehiko Sasaki

**Affiliations:** 1 Department of Urology, Akita University Graduate School of Medicine, Akita, Japan; 2 Research Center for Biosignaling, Akita University, Akita, Japan; 3 Department of Medical Biology, Akita University Graduate School of Medicine, Akita, Japan; 4 Department of Biochemical Pathophysiology/Lipid Biology, Medical Research Institute, Tokyo Medical and Dental University, Tokyo, Japan; 5 Department of Occupational Therapy, Akita University Graduate School of Health Science, Akita, Japan; 6 AMED-CREST, Japan Agency for Medical Research and Development, Tokyo, Japan

**Keywords:** Lipidomics, Prostate cancer

## Abstract

Phosphoinositides (PIPs) participate in many cellular processes, including cancer progression; however, the metabolic features of PIPs associated with prostate cancer (PCa) are unknown. We investigated PIPs profiles in PTEN-deficient prostate cancer cell lines, human prostate tissues obtained from patients with PCa and benign prostate hyperplasia (BPH) specimens using mass spectrometry. In immortalized normal human prostate PNT1B cells, PTEN deficiency increased phosphatidylinositol tris-phosphate (PIP_3_) and decreased phosphatidylinositol mono- and bis-phosphate (PIP_1_ and PIP_2_), consistent with PTEN’s functional role as a PI(3,4,5)P_3_ 3-phosphatase. In human prostate tissues, levels of total (sum of all acyl variants) phosphatidylinositol (PI) and PIP_1_ in PCa were significantly higher than in BPH, whereas PIP_2_ and PIP_3_ contents were significantly lower than in BPH. PCa patients had significantly higher proportion of PI, PIP_1,_ and PIP_2_ with 0–2 double bonds in acyl chains than BPH patients. In subgroup analyses based on PCa aggressiveness, mean total levels of PI with 0–2 double bonds in acyl chains were significantly higher in patients with pathological stage T3 than in those with pathological stage T2. These data indicate that alteration of PIPs level and the saturation of acyl chains may be associated with the development and aggressiveness of prostate cancer, although it is unknown whether this alteration is causative.

## Introduction

Prostate cancer (PCa) is one of the most common malignancies in men worldwide^1^. Epidemiological studies have shown that systematic metabolic disorders and fat-rich diets might increase the risk of developing PCa^2^. Therefore, it is important to understand the contribution of dietary lipids and/or *de novo* lipid synthesis on PCa development. Lipid metabolism and its related molecules play an important role in human cancers, including PCa, by modulating numerous cellular processes^3,4^. Signalling by phosphoinositides (PIPs) using a lipid messenger cascade involving a family of minor acidic phospholipids in cell membranes regulates a substantial number of intracellular proteins with various functions^5–7^. In fact, the phosphatase and tensin homolog (PTEN), which is the phosphoinositide 3-phosphatase, is the most commonly altered gene in PCa, and conditional knockout of this gene in mouse prostates results in the development of PCa^8,9^. Furthermore, other genes encoding PIPs-metabolising enzymes, such as INPP4B and PI3KCA, are known to be associated with PCa progression^10,11^.

In mammalian cells, eight PIPs (PI, PIP_3_, and three regioisomers each of PIP_1_ and PIP_2_) are interconverted and regulated by 19 kinases and 29 phosphatases^5^. Although several studies have been conducted to assess PI levels in PCa^12,13^, comprehensive profiles of PIPs class levels, including PI and its phosphorylated forms such as PIP_1_, PIP_2_ and PIP_3_, remain unelucidated in PCa. PIPs are glycerophospholipids, composed of a glycerol central moiety conjugated to two fatty acid esters (“tail group”) and inositol ring (“head group”)^14^. The “head group” status of these lipids play a key role in biology of PIPs, whereas the lipid tails play a limited role in signaling^14^. Therefore, the expression profile and impact of lipid acyl chains in PCa have remained largely unknown.

Here, we investigate PIPs profiles in PCa using preclinical prostate cells and human PCa tissues using an original method utilising mass spectrometry. To our knowledge, this study is the first to investigate PIPs expression patterns including PIP_1_, PIP_2_ and PIP_3_, as well as their acyl chain profiles, in preclinical prostate cells and clinical PCa specimens. We thereby successfully confirm PIPs changes in PCa using *in vitro* PTEN-driven preclinical PCa models and also show specific features of PIPs levels in human PCa compared with benign prostate tissues.

## Results

### PTEN deficiency increases PIP_3_ levels in the PNT1B normal prostate epithelial cell line

**Figure 1:**
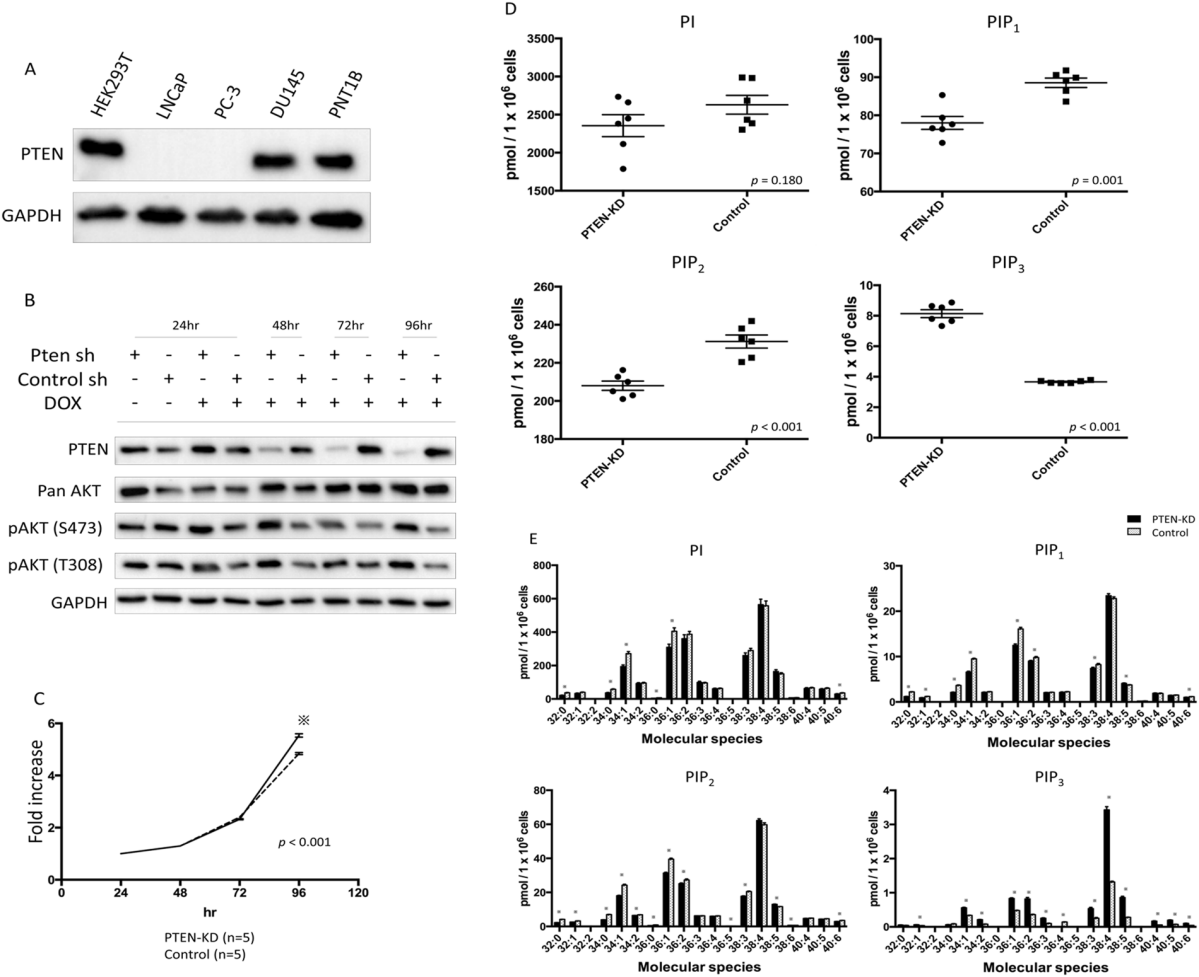
Establishment and characteristics of doxycycline-induced PTEN-KD prostate cells and PIPs profiles of PTEN-KD and control prostate cells. (**A**) PTEN expression in HEK293T cells and various prostate cell lines. Other supporting data were shown in Supplementary Fig. 1. (**B**) PTEN expression and AKT activation in doxycycline-induced PTEN-KD PNT1B and PTEN-WT PNT1B cells (control). Other supporting data were shown in Supplementary Fig. 2. (**C**) Cell proliferation in PTEN-KD and control PNT1B cells. The MTT assay was performed at 24, 48, 72, and 96 h. Cell viability was compared with cells at 24 h. *p < 0.05. (**D**) Total PIPs levels in PTEN-KD and control cells (n = 6) determined using mass spectrometry. The levels are reported as concentration per 1 × 10^6^ cells. The box-dot plot presents the levels of total PIPs in PTEN-KD and control cells. (**E**) PIPs profiles in PTEN-KD and control cells (n = 6) determined using mass spectrometry. The levels are reported as concentration per 1 × 10^6^ cells. Black bars, PTEN-KD cells; white bars, control cells. *p < 0.05.

To assess changes in PIPs levels of prostate cell line associated with the loss of PTEN, which is known to be a tumour suppressor gene as well as a PI(3,4,5)P_3_ 3-phosphatase, we used non-neoplastic human epithelial PNT1B prostate cells that exhibit wild-type PTEN expression, as previously reported (Fig. 1A, Supplementary Fig. 1)^15^. After doxycycline (dox)-inducible PTEN knockdown (KD) in the PNT1B cells, we confirmed lowered PTEN expression and a higher activation level of AKT (S473 and T308) than in the control cells (Fig. 1B, Supplementary Fig. 2). Cell proliferation at 96 h in the PTEN-KD PNT1B cells was significantly higher than that in the control cells (p < 0.001, Fig. 1C).

We then examined the levels of PIPs by a reverse phase LC/MS/MS method that allows quantitation of PI, PIP_1_ (as the sum of PI3P, PI4P, and PI5P), PIP_2_ (as the sum of PI(3,4)P_2_, PI(3,5)P_2_, and PI(4,5)P_2_), and PIP_3_. The most abundant class of PIPs in both PTEN-KD cells and control cells was PI, followed by PIP_2_, PIP_1_, and PIP_3_ (Fig. 1D). The mean level of total PIP_3_ in the PTEN-KD cells was significantly higher than that in the control cells (8.14 pmol/1 × 10^6^ cells vs. 3.67 pmol/1 × 10^6^ cells, p < 0.001), whereas the mean levels of PIP_1_ and PIP_2_ in the PTEN-KD cells were significantly lower than in the control cells (p = 0.001 and p < 0.001, respectively). There was no significant difference in total PI levels between the PTEN-KD and control cells. Since PTEN is a PI (3,4,5) P_3_ 3-phosphatase, increased PIP_3_ and decreased PIP_1_ and PIP_2_ in PTEN-KD cells verified that our method can measure PIPs profiles in prostate cells.

With respect to the composition of cellular PIPs acyl species, the levels of PIPs containing C38:4 (38 carbons:4 double bonds) acyl chains were the highest in both the PTEN-KD and control cells (Fig. 1E). PIPs containing C34:1, C36:1, C36:2, C38:3, and C38:5 acyl chains were also present at relatively high levels in PNT1B cells regardless of PTEN knockdown (Fig. 1E). Approximately 80% of the total cellular PIPs contained the above six acyl chains in both PTEN-KD and control cells. The levels of PIP_3_ containing C32:1, C34:1, C34:2, C36:1, C36:2, C36:3, C38:3, C38:5, C40:4, C40:5, and C40:6 acyl chains in the PTEN-KD cells were significantly higher than in the control cells (Fig. 1E), whereas the level of C36:4 PIP_3_ species was higher in the control cells.

### PIPs profiles in malignant and nonmalignant human prostate tissues

To investigate the PIPs profiles of human prostates, we measured the PIPs levels in prostate tissues obtained from patients with PCa who underwent robot-assisted radical prostatectomy with those from patients with benign prostate hyperplasia (BPH) treated with transurethral resection or holmium laser enucleation of the prostate.

**Figure 2:**
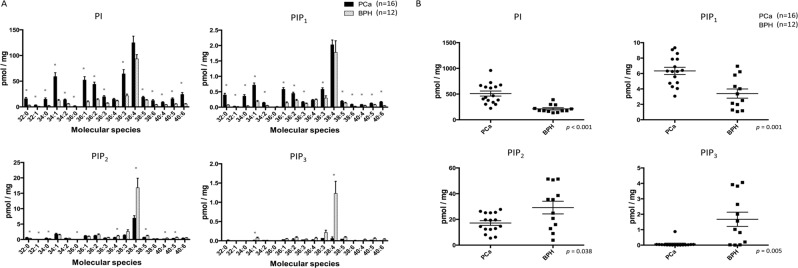
PIPs levels in human prostate tissues. (**A**) PIPs acyl chain profiles in PCa and benign prostate hyperplasia tissues obtained from surgical patients. Results are presented as the mean ± SE (pmol/mg) of data from 16 PCa and 12 benign prostate hyperplasia samples. Black bars, prostate cancerous tissues; white bars, benign prostate tissues. *p < 0.05. (**B**) Comparison of total PIPs in prostate samples obtained from patients with cancer or benign prostate hyperplasia. The box-dot pot presents the levels of total PIPs in human prostate cancer and benign prostate hyperplasia.

**Figure 3:**
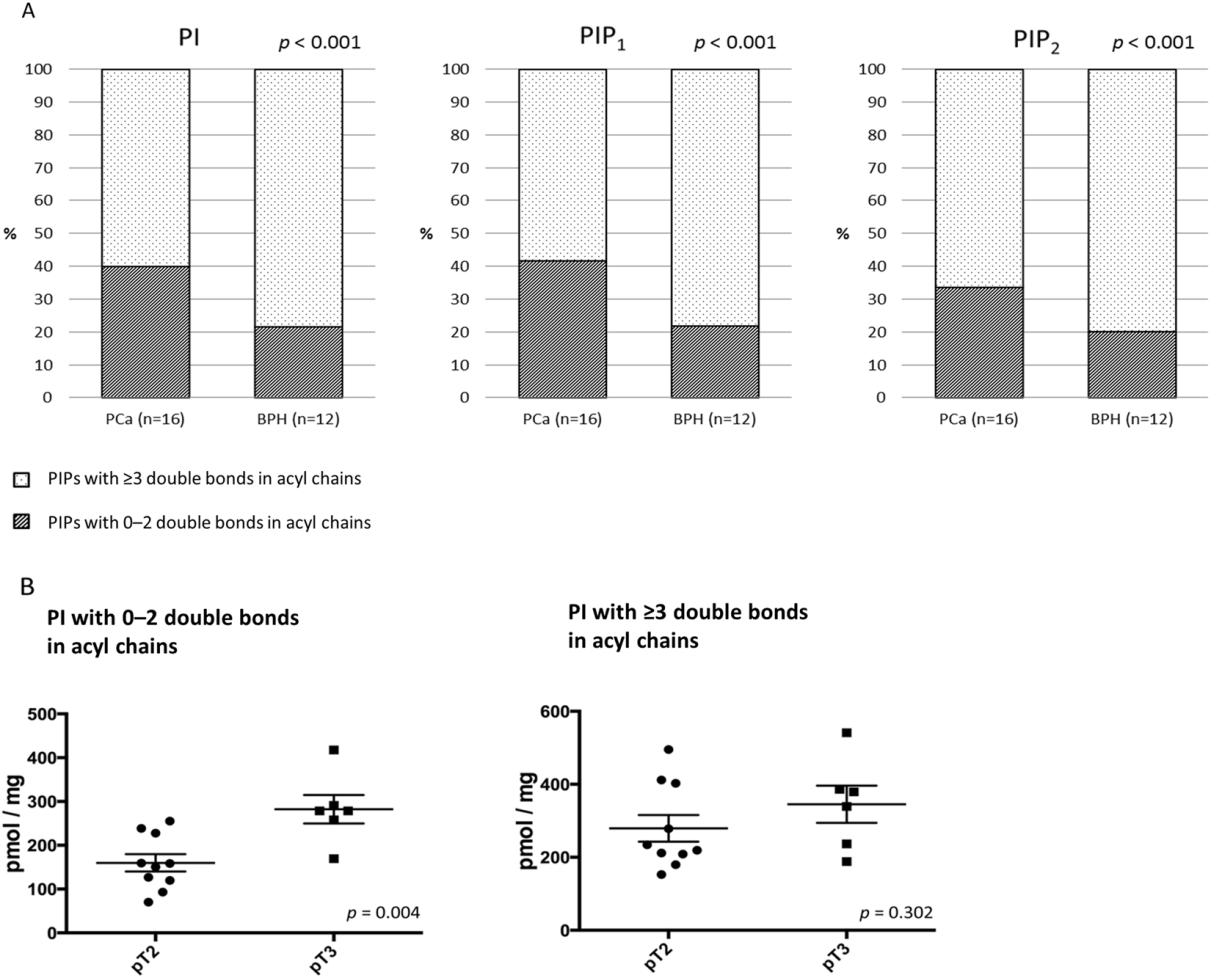
Saturation status of PIPs acyl chains of human prostate tissues. (**A**) All patients were subcategorized into two groups based on the number of double bonds in acyl chains. Percentages of each PIP with 0–2 double bonds in acyl chains (red) or with ≥3 double bonds in acyl chains (blue) in PCa (n = 16) and benign prostatic hyperplasia (n = 12) tissues are shown. The *p* values were determined using a chi-square test. (**B**) Comparison of PI levels dichotomised by saturation status in prostate tissues based on pathological T stage. The box-dot plot presents the levels of PI in human prostate cancer.

Figure 2A demonstrates the levels of each PIPs class in human PCa and BPH tissues. The most abundant PIPs in both tissues were PI, followed by PIP_2_, PIP_1_ and PIP_3_, which was consistent with the order observed in the human prostate cell line (Fig. 1D). In human prostate tissues, the mean levels of total PI and PIP_1_ in the PCa tissues were significantly higher than those in the BPH tissues (PI: 510.4 ± 49.8 pmol/mg vs. 213.2 ± 22.0 pmol/mg, *p* < 0.001; PIP_1_: 6.3 ± 0.5 pmol/mg vs. 3.4 ± 0.6 pmol/mg, *p* = 0.001; Fig. 2B). In contrast, the mean levels of total PIP_2_ and PIP_3_ in the PCa tissues were significantly lower than in the BPH tissues (17.2 ± 1.9 pmol/mg vs. 29.2 ± 4.9 pmol/mg, p = 0.038; 0.07 ± 0.05 pmol/mg vs. 1.67 ± 0.46 pmol/mg, p = 0.005; Fig. 2B). With respect to the acyl chain pattern of PIPs in human prostate tissues, the C38:4 acyl chain was the most abundant in PIPs from both cancerous prostate and BPH tissues (Fig. 2A). As was the case with the prostate cancer cell line, PIPs containing C34:1, C36:1, C36:2, and C38:3 acyl chains were also abundant in both cancerous prostate and BPH tissues (Fig. 2A). Intriguingly, we found that the PCa specimens exhibited a significantly higher proportion of total PI, PIP_1_, and PIP_2_ with 0–2 double bonds in acyl chains and lower proportion of total PI, PIP_1_, and PIP_2_ with ≥3 double bonds in acyl chains compared with that in the BPH samples (Fig. 3A, Supplementary Table 1). The levels of PIP_3_ acyl species in most of the PCa tissues were undetectable (Fig. 2A).

Finally, we assessed the difference in PIPs profiles between PCa tissues in terms of cancer aggressiveness based on subgroup categories, including preoperative serum prostate-specific antigen (PSA), Gleason grading group, and pathological T stage (Table 1). The characteristics of the prostate cancer patients investigated in this study are described in Supplementary Table 2. There were no significant differences in levels of total PI, PIP_1_, or PIP_2_ among dichotomised groups based on preoperative PSA level and biopsy Gleason grading (Table 1). With respect to the relationship between pathological stage and PIPs levels, the amount of total PI tended to be higher in patients with pT3 than those with pT2 (p = 0.065). Moreover, the quantity of PI with 0–2 double bonds in acyl chains was significantly higher in patients with pT3 than that in patients with pT2 (p = 0.004, Fig. 3B).These results demonstrate that an increased proportion of PI with 0–2 double bonds in acyl chains to that with ≥3 double bonds in acyl chains may be a hallmark of more aggressive cancerous prostate tissues.

**Table 1:** Subgroup analyses of PIPs profiles in prostate cancer tissues based on clinicopathological findings in patients with prostate cancer who underwent radical prostatectomy.

Total PIPs (pmol/mg, mean ± SE)	Preoperative prostate-specific antigen			Gleason Grading group			Pathological T stage		
	Low PSA (n = 8)	High PSA (n = 8)	*p* value	≦4 + 3 (n = 11)	4+4 ≦ (n = 5)	*p* value	pT2 (n = 10)	pT3 (n = 6)	*p* value
PI	575.3 ± 75.1	445.6 ± 61.6	0.203	494.0 ± 61.6	546.5 ± 91.9	0.642	439.9 ± 55.4	627.9 ± 79.1	0.065
PIP_1_	6.0 ± 0.7	6.7 ± 0.6	0.491	6.6 ± 0.5	5.7 ± 0.9	0.366	6.2 ± 0.6	6.5 ± 0.7	0.765
PIP_2_	15.5 ± 3.1	18.9 ± 2.0	0.370	17.8 ± 2.2	15.8 ± 3.7	0.630	16.3 ± 2.1	18.7 ± 3.6	0.547
PI with 0–2 double bonds in acyl chains	232.3 ± 34.5	179.3 ± 28.4	0.256	198.8 ± 28.6	221.1 ± 39.6	0.664	160.0 ± 19.8	282.2 ± 32.6	0.004
PI with ≥3 double bonds in acyl chains	342.4 ± 47.4	265.7 ± 34.0	0.210	294.5 ± 33.6	325.0 ± 66.0	0.653	279.4 ± 36.6	345.2 ± 50.9	0.302
PIP_1_ with 0–2 double bonds in acyl chains	2.5 ± 0.4	2.9 ± 0.4	0.429	2.8 ± 0.3	2.3 ± 0.6	0.353	2.4 ± 0.3	3.1 ± 0.5	0.265
PIP_1_ with ≥3 double bonds in acyl chains	3.5 ± 0.5	3.8 ± 0.3	0.662	3.8 ± 0.3	3.4 ± 0.5	0.496	3.8 ± 0.4	3.5 ± 0.3	0.571
PIP_2_ with 0–2 double bonds in acyl chains	5.2 ± 1.1	6.3 ± 0.9	0.462	5.9 ± 0.8	5.4 ± 1.6	0.747	5.0 ± 0.7	7.0 ± 1.4	0.169
PIP_2_ with ≥3 double bonds in acyl chains	10.2 ± 2.2	12.6 ± 1.3	0.362	11.9 ± 1.6	10.4 ± 2.2	0.593	11.2 ± 1.5	11.7 ± 2.4	0.878

## Discussion

This is the first report demonstrating the changes in PIPs, including PI, PIP_1_, PIP_2_ and PIP_3_, at the acyl species level in human BPH and PCa. Using a mass spectrometry-based approach, we found that deletion of the PIP_3_ 3-phosphatase, PTEN, in immortalised benign prostate cells resulted in increases in various acyl species of PIP_3_, which validated the applicability of the method to biological samples. The measurements of PIPs in human prostate samples revealed that PCa tissues had higher PI and PIP_1_ levels and lower levels of PIP_2_ and PIP_3_ compared with BPH. In addition, PIPs in cancerous prostate tissues had a higher proportion of PIPs with 0–2 double bonds in acyl chains and a lower proportion of PIPs with ≥3 double bonds in acyl chains than those in BPH. In addition, PCa with capsular or seminal invasion had higher PI with 0–2 double bonds in acyl chains compared with that in organ-confined PCa. These results suggest that PIPs class levels and their acyl chain profiles are altered in cancerous prostate tissues and in aggressive phenotypes of PCa in particular.

There have been several studies investigating phospholipid levels in human prostate tissues^12,13,16^. A study using high-resolution matrix-assisted laser desorption/ionisation imaging mass spectrometry demonstrated that PI (18:0/18:1), PI (18:0/20:3), and PI (18:0/20:2) were significantly elevated in PCa tissues relative to benign epithelium and thus proposed a biomarker algorithm using PI profiles to distinguish cancerous from benign epithelia^13^. These results are concordant with those of the present study showing that the levels of PI C36:1 and PI C38:3 are significantly higher in PCa than in the BPH. The present study also shows that the elevation of PI level is not specific to a particular PI acyl species, since the total PI level, including several PI acyl species mentioned above, was higher in cancerous prostate tissues. In addition to the PI status in human prostates, the present study is the first to successfully evaluate its phosphorylated derivatives, including PIP_1_, PIP_2_ and PIP_3_, and showed that the level of total PIP_1_, as well as the level of total PI, is significantly higher in prostate cancer tissues than in BPH. In contrast, the level of PIP_2_ and PIP_3_ is lower in prostate cancer tissues than in BPH. Since prostate cancer is known to exhibit a higher rate of PTEN alteration, we speculated that PIP_3_ levels were elevated in PCa tissues because PCa is known to frequently contain a class I PI3K alteration. The reason for the PIP_3_ level decrease in the PCa tissues remains unclear; however, homozygous deletions of the PTEN locus occurred in just 15% of samples in a comprehensive molecular analyses of 333 primary prostate cancers using the TCGA database^17^, and a substantial amount of molecular heterogeneity has been observed in clinically extracted prostate cancer tissues. In addition, although our preliminary results showed that there was no significant association between PTEN immunoreactivity scores and PIPs levels in the tumors (Supplementary Fig. 3), future studies focusing on the interaction between PIPs class levels and mutation status/expression of PTEN using a larger number of patients are needed.

Of note, we found a higher proportion of PIPs with 0–2 double bonds in acyl chains and a lower proportion of PIPs with ≥3 double bonds in acyl chains in human PCa. In the current study, we could not distinguish two acyl chains in each PIP separately, which means that the clear classification of acyl chains into saturated/monounsaturated fatty acid (SMFA) and polyunsaturated fatty acids (PUFA) was not performed. However, we confirmed the higher levels of SMFA and lower levels of PUFA in PIPs of cancerous tissues, after excluding C34:2 and C36:2 acyl species from the statistical analysis (Supplementary Fig. 4). Accordingly, our study revealed that the lipid saturation in PIPs increased in PCa tissues, especially in advanced PCa.

In a comprehensive lipidomic analysis of 267 human breast tissues, Hilvo *et al*. demonstrated that saturated-fat-containing phosphatidylcholines were elevated in tumors compared with normal breast tissues and associated with clinical outcomes^18^. Guo *et al*. conducted a lipidomic study of 134 tissue samples from six different types of malignancies (breast, lung, colorectal, oesophageal, gastric, and thyroid) using matrix-assisted laser desorption/ionization Fourier-transform ion cyclotron resonance mass spectrometry and demonstrated that monounsaturated fatty acids and monounsaturated phosphatidylcholines levels increased significantly in the cancer microenvironment compared with that in the adjacent normal tissue^19^. In PCa, a study of phospholipid composition by ESI-MS/MS showed that tumours with increased expression of the lipogenic enzyme FASN had a consistent increase in SMFA acyl chains and decrease in PUFA acyl chains of phosphatidylcholine in prostate tumour tissues compared with matching normal tissues^12^. Furthermore, PUFAs were easily influenced by lipid peroxidation, which affects oxidative stress-induced cell death in PCa LNCaP cells. The present study is the first to reveal a high level of three PIPs classes (PI, PIP_1_, and PIP_2_) containing 0–2 double bond acyl chains in prostate cancer tissues, and especially in aggressive prostate cancer, and along with these other lines of evidence, suggests that cancerous tissues contain elevated levels of phospholipids with SMFA acyl chains and that the SMFAs/PUFAs balance in tumours may be associated with cancer development and progression. In fact, a study using human glioblastoma and breast cancer cell lines to assess the impact of PUFA and SMFA supplementation on the growth of cancer cells showed that PUFA supplementation inhibited cancer cell growth, whereas SMFA enhanced their proliferation^20^. Moreover, related enzymes such as Stearoyl-CoA desaturase (SCD; a rate-limiting enzyme in the biosynthesis of monounsaturated fatty acids) are highly expressed in human prostate and breast cancers^21,22^, whereas a lower level of delta-6-desaturase (the main determinants of PUFA levels) has been observed in breast tumours compared with normal tissues^23^. The actual SMFA and PUFA profiles in two acyl chains of each PIP in PCa tissues should be evaluated in future studies to clarify key lipogenic enzymes in prostate cancer aggressiveness. Taken together, these lipogenic features in cancer, including PCa, provide specific biomarkers for the detection of aggressive phenotypes of prostate cancer and suggest that these enzymes are potential targets for the exploration of a novel treatment strategy of lipid desaturation to overcome treatment resistance and the aggressive phenotype of PCa.

This study has several limitations. Regarding *in vitro* study, we found a time lag between the difference in PTEN expression and the impact of cell proliferation. We speculated that differences in the expression and/or activation of down-stream targets of PTEN are also important in modulating cell proliferation. In fact, the activation of AKT in PTEN-KO cells at 96 h was higher than that at 72 h. Moreover, other potential downstream targets, such as p27 and Bad, may influence the difference in cell proliferation. In human study, we did not assess saturation status of other membrane lipids other than PIPs in this study, and it would be intriguing to know whether or not this higher lipid saturation status is specific to PIPs. We also did not distinguish between regioisomers of PIPs, for example, PI(3,4)P_2_ and PI(4,5)P_2_. The levels of each regioisomer and its functional impact on PCa should be clarified in a future study. Additionally, information on the dynamic changes of the PIPs profile in PCa remains elusive because we only measured the PIPs profile at specific time points in preclinical and clinical PCa. Furthermore, in humans, we only compared PIPs levels in pathogenic prostate tissue with BPH or PCa. Therefore, further study is needed to investigate the difference in PIPs profiles in other prostate tissues, including normal prostate from healthy subjects. Finally, we cannot assess the pure expression profiles of prostate epithelial cells because the location of cancerous regions is quite heterogeneous in the prostate, which poses difficulties for the isolation of cancerous cells. The expression profiles in cancer cell and the cancer stromal environment has therefore yet to be clarified.

In conclusion, our study successfully measured PIPs class levels and acyl chain profiles in preclinical prostate cells and clinical PCa specimens. PTEN deletion resulted in increased PIP_3_ levels in prostate cells with declines in PIP_2_ and PIP_1_. The total levels of PIPs and acyl chain components differed in human PCa compared with BPH, and advanced PCa possessed specific PIPs profiles. Evaluating PIPs profiles in PCa may provide an understanding of the detailed mechanism of PCa initiation and progression as well as novel biomarkers for screening and prognostication. In particular, high PI/PIP_1_ and the targeting of saturation status, respectively, have the potential to predict cancer progression and become a novel treatment strategy for PCa.

## Methods

### Cell lines

Human PCa cells (DU-145, LNCaP, and PC-3) and the Human Embryonic Kidney 293 cell (HEK-293) lines were obtained from the American Type Cell Culture Collection (Manassas, VA, USA) or RIKEN BioResource Center (Tsukuba, Japan). The normal prostate epithelial cell line, PNT1B, was provided by Professor N. Maitland (York, UK). The cell lines were cultured in RPMI-1640 containing 10% foetal bovine serum (FBS) in a 5% CO_2_ atmosphere of a humidified incubator at 37 °C.

### Human prostate tissues

Fresh human prostate tissues were obtained from patients who underwent radical prostatectomy without any preoperative hormonal or chemotherapeutic treatment at Akita University Hospital between 2015 and 2017^24^. All patients were histologically diagnosed with localised PCa and had large, visible suspected tumour regions upon preoperative MRI imaging. The patients’ characteristics are described in Supplementary Table 2. The prostates were first coronally dissected into four pieces at even intervals. Slices of the prostates, including ≥50% of the cancer regions with pathological diagnosis of their mirror images, were then snap-frozen in liquid nitrogen and stored at −80 °C until needed. All slides were reviewed by a pathologist (T.Y.), who was blinded with respect to each patient’s clinical background^24^. Human benign prostate tissues were obtained from patients treated by transurethral resection of the prostate or by holmium laser enucleation of the prostate (HoLEP) at hospitals affiliated with Akita University School of Medicine. The resected pieces of the prostates were snap-frozen in liquid nitrogen and stored at −80 °C until needed. This study was conducted in accordance with the Declaration of Helsinki, and the protocol was approved by the Institutional Review Board of the Akita University School of Medicine (IRB #1034). Written informed consent was obtained from each participant prior to obtaining prostate tissues.

### Antibodies

Monoclonal antibodies against PTEN, pan-AKT, phospho-AKT (S473), and phospho-AKT (T308) were purchased from Cell Signaling Technology (Danvers, MA, USA).

### Western blotting

Prostate cells were homogenised in lysis buffer (50 mM Tris–HCl, pH 8.0, 100 mM NaCl, 1 mM EDTA, 1% Triton X-100, 1 mM DTT, 1 mM Na3VO4, 30 mM sodium pyrophosphate, 50 mM sodium fluoride, and protease inhibitor cocktail [Roche Applied Science, Mannheim, Germany]). After centrifugation at 20,000* g* for 15 m, supernatants (10 µg total protein) were subjected to standard SDS-PAGE and immunoblotting as previously described^25^.

### Establishment of a doxycycline-inducible PTEN-knockdown PNT-1B cell line

The sh-RNA plasmid against PTEN was purchased from OpenBiosystems (USA). Lentiviral production was performed per the supplied protocol and as used in a previous study^25^. After a 72 h incubation at 37 °C, lentivirus-containing supernatant was harvested and applied to PNT1B cells. Once stable in culture, puromycin selection was initiated at a concentration of 1 μg/mL. To induce sh-RNA expression, doxycycline was added to culture medium at a concentration of 1 μg/mL and replaced every 24 h^26^.

### Cell proliferation assay

PNT1B cells were seeded into 6-well plates at a density of 3 × 10^4^ per well in culture medium containing 10% FBS. Cell proliferation assays were conducted using a non-radioactive 3-(4,5-dimethylthiazol-2-yl)-2,5-diphenyltetrazolium bromide (MTT)-based Cell Proliferation Kit (Roche Life Sciences, Branford, CT, USA) according to the manufacturer’s instructions. Absorbance was measured using an ELISA reader (Bio-Rad Laboratories, Inc., Hercules, CA, USA). Proliferation assays were performed in triplicate.

### Histological analysis

Human prostate tissues were fixed in 10–20% formalin neutral buffer solution and embedded in paraffin. Sections (3–5 μm) were cut and stained with hematoxylin and eosin in accordance with standard procedures.

### Preparation of samples for LC-MS/MS

Tissues (10 mg) or cultured cells (1 × 10^7^) were minced on ice in 700 μL of buffer (10 mM Tris–HCl, pH 7.4, 20 mM NaCl, 1 mM EDTA, 1% Triton X-100, and protease inhibitor cocktail) and incubated at 4 °C for 20 min. The lysates were transferred to glass tubes and mixed with 700 μL of methanol containing 1 nmol of 8:0/8:0 PI(4,5)P2 (Avanti Polar Lipids, Inc., Alabaster, AL, USA) to prevent adsorption of cellular phosphoinositides to the glassware during the procedure. After the addition of 17:0/14:1-phosphatidylinositol, 17:0/20:4-phosphatidylinositol 4-monophosphate, 17:0/20:4-phosphatidylinositol 4,5-bisphosphate, and 17:0/20:4-phosphatidylinositol 3,4,5-trisphosphate (Avanti Polar Lipids) as internal/surrogate standards (10 pmol each), the lysates were subjected to lipid extraction with methanol/2 M HCl/chloroform (1:2:4, v/v/v). To condense anionic phospholipids, the resultant organic phase was loaded onto a diethylaminoethyl (DEAE)-cellulose column (SantaCruz Biotechnology, Dallas, TX, USA). The column was then washed sequentially with 3 mL of chloroform/methanol (1:1) and 2 mL of chloroform/methanol/saturated (28%) ammonia/glacial acetic acid (200:100:3:0.9), and acidic phospholipids (mostly phosphoinositides and phosphatidylserine) were eluted with 1.5 mL of chloroform/methanol/12 M HCl/water (12:12:1:1). The eluates were mixed vigorously for 2 min with 850 μL of 125 mM NaCl followed by centrifugation at 3,000 *g* for 3 min at room temperature. The lower phase was subjected to a methylation reaction using trimethylsilyl diazomethane (Tokyo Chemical Industry, Co. Ltd., Tokyo, Japan) according to the method of Clark *et al*.^27^. Extracts of the resultant derivatives were taken to dryness under a stream of nitrogen, and the residues were dissolved in 36 μL of methanol/70% ethylamine/water (100:0.065:33).

### Mass spectrometry

An Ultimate 3000 LC system (Thermo Fisher Scientific) connected in tandem to a TSQ Vantage triple stage quadrupole mass spectrometer (Thermo Fisher Scientific) in the positive-ion mode was used for LC-MS/MS analysis. The methylated phospholipids (20 μL/injection) were separated on an InertSustainBio C18 column (GL Sciences) using the following solvent gradient: 0–1 min hold 70% A/30% B, 1–3 min constant gradient to 90% A/10% B, 3–7.5 min constant at 90% A/10% B, and 7.5–13 min 30% A/70% B, where mobile phase A was acetonitrile/water/70% ethylamine (800:200:1.3) and mobile phase B was isopropanol/acetonitrile/70% ethylamine (800:200:1.3). The injection volume was 20 μL, and the flow rate was set at 220 μL/min. Multiple reaction monitoring was employed for quantitation of phosphoinositide species using a pre-set list of the mass to charge ratio (m/z) values. The parent ions (the number of methylation site(s) for PI, PIP_1_, PIP_2_ and PIP_3_ were 1, 3, 5 and 7, respectively) were selected in the first quadrupole and subjected to fragmentation with collision-induced dissociation followed by monitoring of the product ions (diacylglycerols with different fatty acyl compositions)^28^. Peak areas of individual species were normalised to the internal/surrogate standards possessing a heptadecanoyl moiety (see “Preparation of samples for LC-MS/MS”). PIPs levels were considered to be zero if the levels under detection limits.

### Statistical analysis

All values are presented as mean ± SE. Unpaired Student’s *t* and Mann–Whitney tests were used to compare differences. Chi-square test is used to examine the association between two categorical variables. Differences were considered significant if p values were < 0.05. All statistical analyses were performed using SPSS ver. 22 statistical software (IBM Corp., NY, USA).

## Supplementary information

supplementary
