# Supplementary material for: Increased fatty acyl saturation of phosphatidylinositol phosphates in prostate cancer progression
Source: Sci Rep. 2019 Sep 13;9:13257. doi: 10.1038/s41598-019-49744-3 (PMC6744559; doi:10.1038/s41598-019-49744-3)
Supplement: Supplementary file 1 — supplementary [file 41598_2019_49744_MOESM1_ESM.docx]

Increased fatty acyl saturation of phosphatidylinositol phosphates in prostate cancer progression

Atsushi Koizumi^1,6^, Shintaro Narita^1,6^, Hiroki Nakanishi^2,6^, Masaki Ishikawa^3^, Satoshi Eguchi^3^, Hirotaka Kimura^3^, Shunsuke Takasuga^3^, Mingguo Huang^1,6^, Takamitsu Inoue^1,6^, Junko Sasaki^3,4,6^, Toshiaki Yoshioka^5^, Tomonori Habuchi^1,6^, and Takehiko Sasaki^3,4,6^

^1^Department of Urology, Akita University Graduate School of Medicine, Akita, Japan

^2^Research Center for Biosignaling, Akita University, Akita, Japan

^3^Department of Medical Biology, Akita University Graduate School of Medicine Akita,

Japan

^4^Department of Biochemical Pathophysiology/Lipid Biology, Medical Research Institute, Tokyo Medical and Dental University, Tokyo, Japan

^5^Department of Occupational Therapy, Akita University Graduate School of Health Science, Akita, Japan

^6^AMED-CREST, Japan Agency for Medical Research and Development

Supplementary Figure1: Fig Gel images of the western blot analysis shown inFigure1A

1 2 3 4 5

PTEN


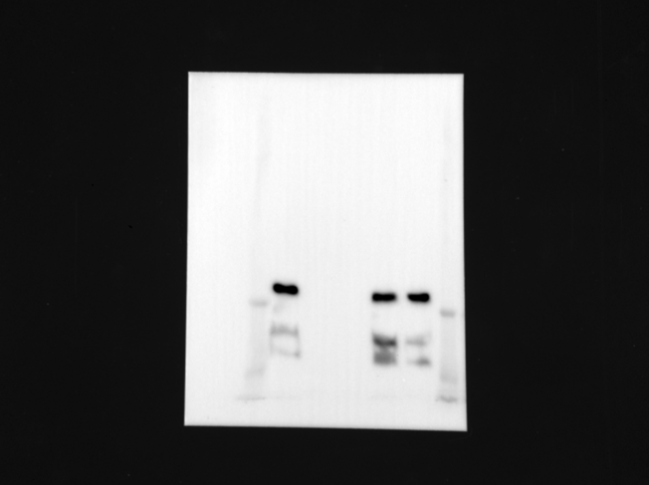


-50kDa

GAPDH


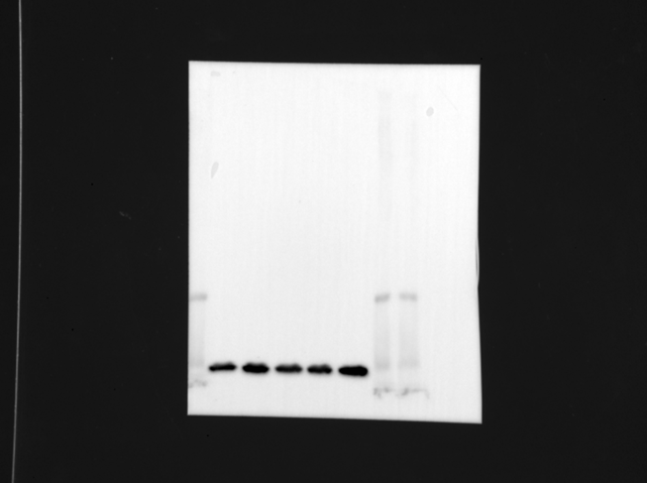


-50kDa

-37kDa

1 HEK293T

2 LNCaP

3 PC-3

4 DU145

5 PNT1B

1 2 3 4 5

Supplementary Figure 2: Fig Gel images of the western blot analysis shown inFigure1B

1 2 3 4 5 6 7 8 9 10


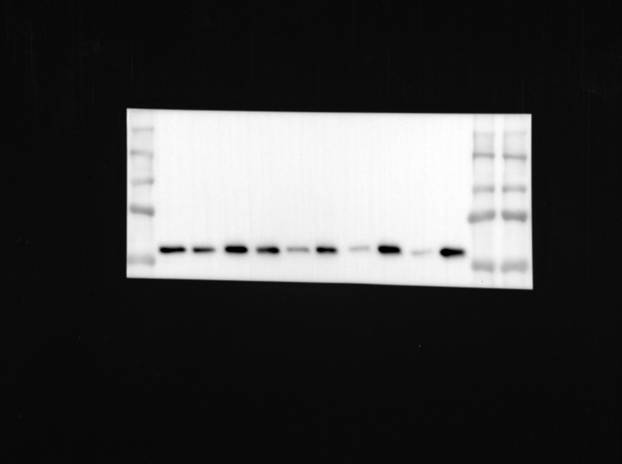


PTEN

-200kDa


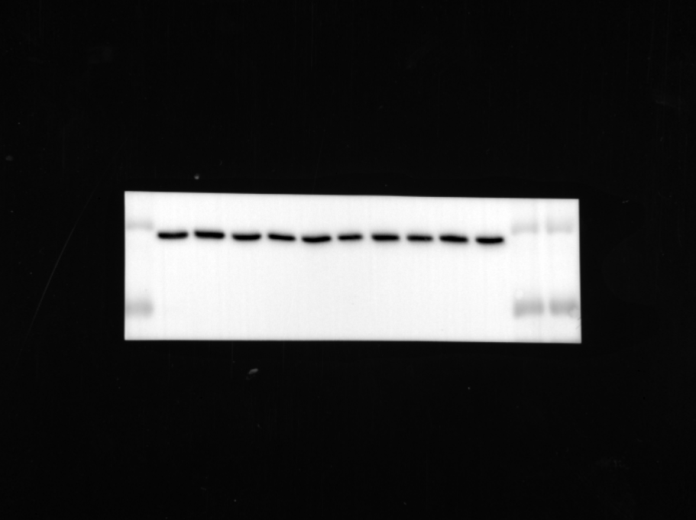

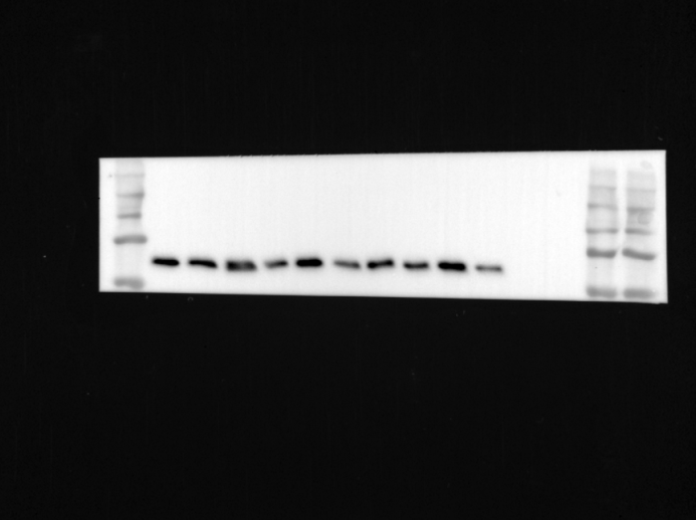

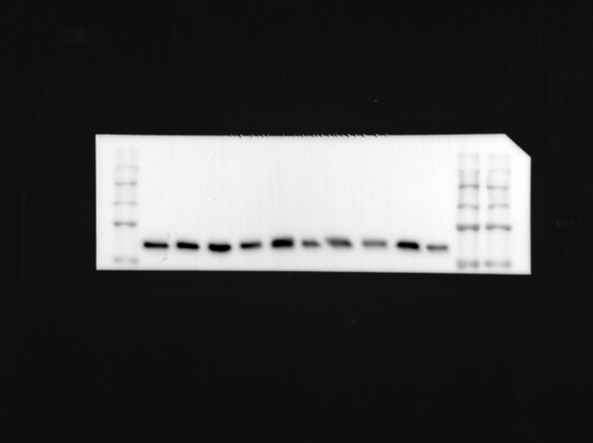

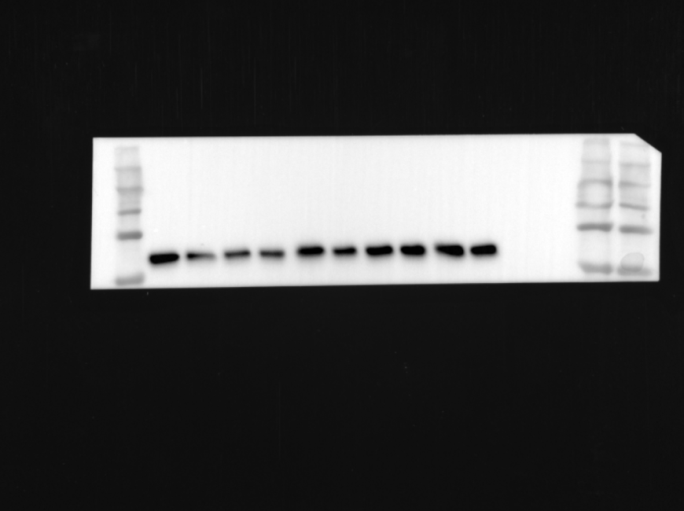


-50kDa

-37kDa

-50kDa

-25kDa

-150kDa

-100kDa

-75kDa

-150kDa

-100kDa

-75kDa

GAPDH

pAKT (T308)

pAKT (S473)

Pan AKT

-150kDa

-100kDa

-200kDa

-75kDa

-50kDa

-50kDa

-75kDa

-100kDa

-150kDa

1 Pten sh(+) Control sh(-) DOX(-) 24hr

2 Pten sh(-) Control sh(+) DOX(-) 24hr

3 Pten sh(+) Control sh(-) DOX(+) 24hr

4 Pten sh(-) Control sh(+) DOX(+) 24hr

5 Pten sh(+) Control sh(-) DOX(+) 48hr

6 Pten sh(-) Control sh(+) DOX(+) 48hr

7 Pten sh(+) Control sh(-) DOX(+) 72hr

8 Pten sh(-) Control sh(+) DOX(+) 72hr

9 Pten sh(+) Control sh(-) DOX(+) 96hr

10 Pten sh(-) Control sh(+) DOX(+) 96hr

1 2 3 4 5 6 7 8 9 10

1 2 3 4 5 6 7 8 9 10

1 2 3 4 5 6 7 8 9 10

1 2 3 4 5 6 7 8 9 10

Supplementary Figure 3: Relationship between PTEN immunoreactivity scores and PIP profiles in human prostate cancer


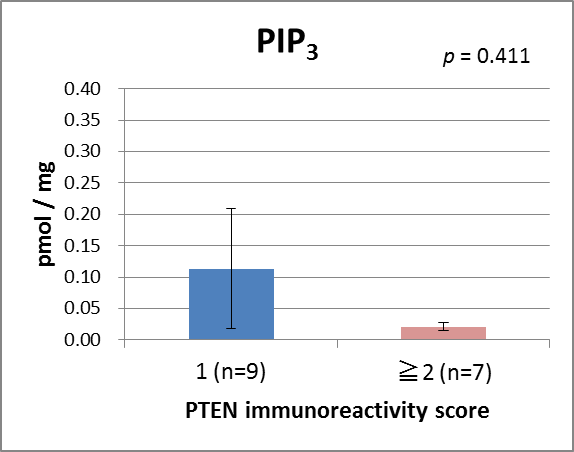

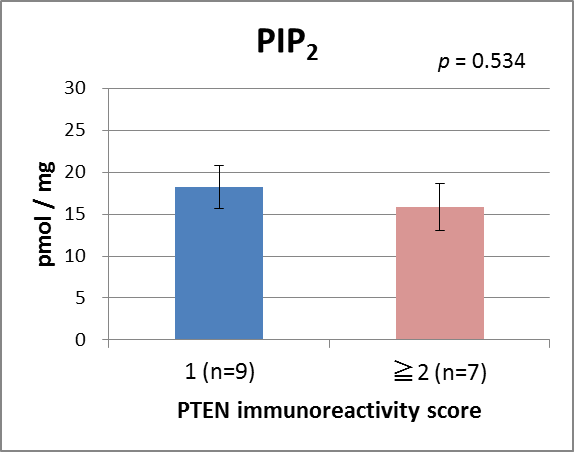

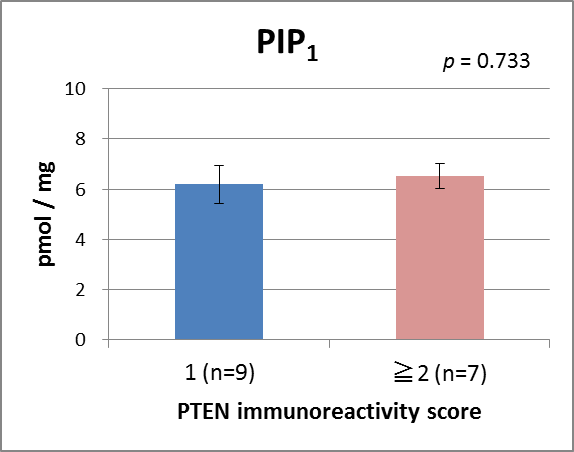

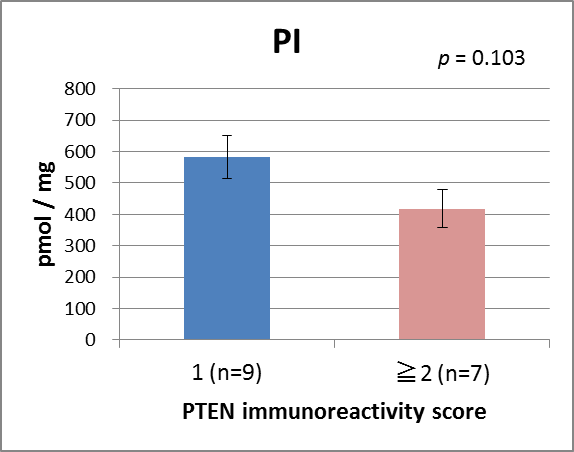


Supplementary Figure 4: Saturation status of PIP acyl chains excluding C34:2PIPs and C36:2PIPs in human prostate tissues. A. Proportion of SMFA and PUFA acyl chains in PIPs in human prostate tissues. B. Comparison of PI levels dichotomized by saturation status in human prostate tissues based on pathological T stage.

A.


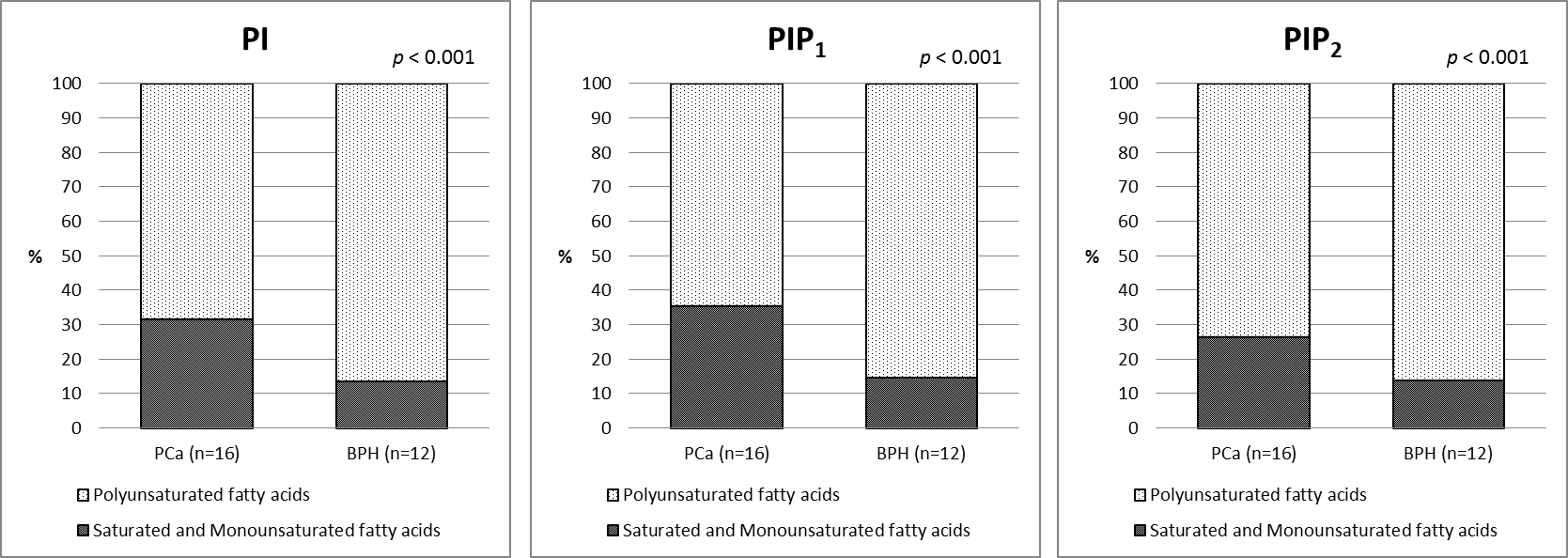


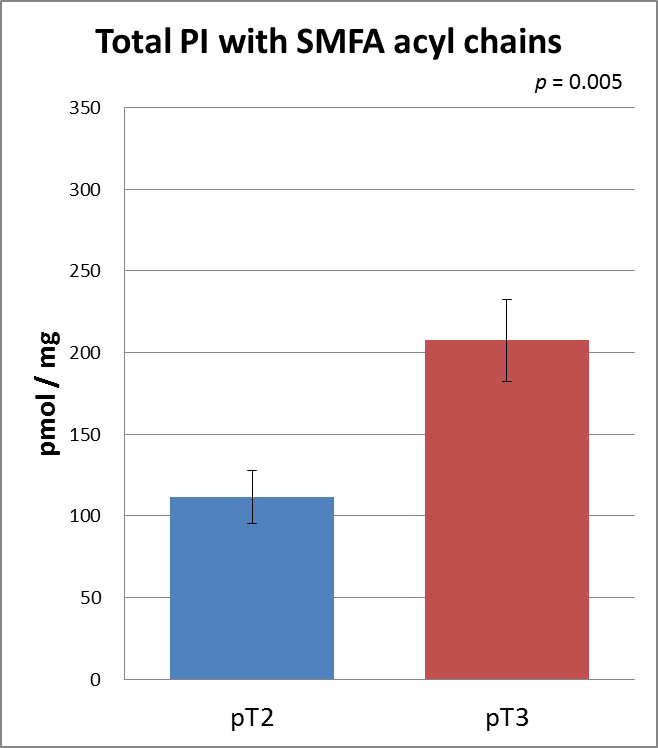

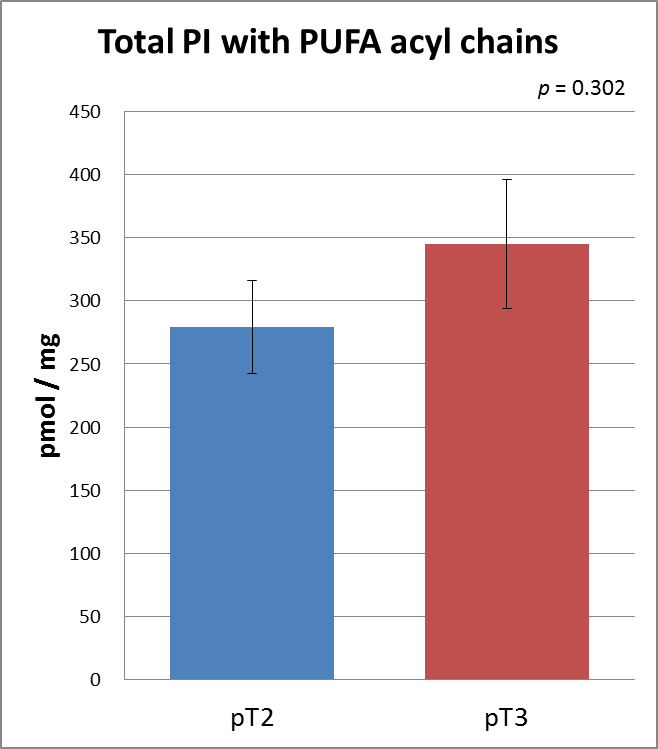
B.
